# PCBs Are Endocrine Disruptors: Mixture Affects Reproductive Development in Female Mice

**Published:** 2006-06

**Authors:** Victoria McGovern

Polychlorinated biphenyls (PCBs) are a broad group of chemicals that includes 209 aromatic chlorinated hydrocarbons used for products ranging from fluorescent light fixtures to coolant fluids inside parts of consumer electronics. Short-term exposure to large amounts of PCBs can cause liver damage; the effects of smaller concentrations can be more subtle, affecting the reproductive development of children of exposed mothers. But are these compounds actually endocrine disruptors? New work by researchers at the Mount Sinai School of Medicine confirms that they can be, and for the first time connects a molecular mechanism to the lifelong phenotypic changes seen after exposure to environmental estrogens **[*EHP* 114:898–904; Ma and Sassoon]**.

Sold predominantly as mixtures under the trade names Aroclor and Pyranol, PCBs have been banned in the United States since 1977 and are out of use or highly restricted throughout much of the world. These compounds, which are fat-soluble and structurally similar to DDT, are highly stable: PCBs made and used for nearly 50 years before the ban remain in the environment and are found throughout the food chain, including in human tissues and breast milk.

The researchers tested the hypothesis that PCBs are endocrine disruptors by comparing their effects on expression of *Wnt7a* in mice. Down-regulation of *Wnt7a* is a known factor in the reproductive deficits found in mice exposed to diethylstilbestrol (DES), a synthetic estrogen once used to supplement estrogen levels in pregnant women. After exposure to DES, development of the reproductive system is impaired when production of the regulatory protein coded by *Wnt7a* is temporarily squelched. A passing loss of appropriate regulation in very young animals leads to reproductive changes—for example, in the number of uterine glands or the thickness of the myometrium, the uterus’s muscular outer layer—that give way to more pronounced changes through life. This suggests that exposed organisms are sent down an abnormal developmental path from which they cannot return.

Using *in situ* hybridization, immunohistochemistry, and quantitative reverse-transcriptase polymerase chain reaction, the authors showed that the effects of environmentally relevant levels of Aroclor 1254, a commercial mix of PCBs, were similar but not identical to those of low levels of DES. Both led to decreases in expression of the *Wnt7a* regulatory gene and showed qualitative changes in uterine development like those described above. The authors also demonstrated that measurement of *Wnt7a* provides a molecular tool that lays to rest any doubt that PCBs are endocrine disruptors.

The authors further showed that genetic makeup matters in terms of vulnerability to endocrine disruption. Mice with only one good copy of the *Wnt7a* gene were more sensitive to DES or PCBs than mice with both copies intact, providing a concrete example of how gene variation can leave some individuals more susceptible than others to the effects of PCB exposure.

